# Efficacy of the combination of amphotericin B and echinocandins against *Candida auris in vitro* and in the *Caenorhabditis elegans* host model

**DOI:** 10.1128/spectrum.02086-23

**Published:** 2023-11-29

**Authors:** Ainara Hernando-Ortiz, Elena Eraso, Nerea Jauregizar, Piet W.J. de Groot, Guillermo Quindós, Estibaliz Mateo

**Affiliations:** 1 Department of Immunology, Microbiology and Parasitology, Faculty of Medicine and Nursing, University of the Basque Country (UPV/EHU), Bilbao, Spain; 2 Department of Pharmacology, Faculty of Medicine and Nursing, University of the Basque Country (UPV/EHU), Bilbao, Spain; 3 Regional Center for Biomedical Research, University of Castilla-La Mancha, Albacete, Spain; University of Lagos, Lagos, Nigeria

**Keywords:** Candidiasis, antifungal activity, drug combinations, *Caenorhabditis elegans* model, *Candida auris*, amphotericin B, echinocandins, synergy

## Abstract

The multidrug-resistant *Candida auris* is a global health emergency, being responsible for outbreaks of invasive candidiasis worldwide. Limited effective therapeutic options make it difficult to treat this emerging pathogen. In this context, combinations of different antifungal drugs are considered a promising therapeutic alternative. The aim of this work was to analyze the antifungal activity of combinations of amphotericin B and echinocandins against five clinical blood isolates of *C. auris*. One of these isolates showed an aggregative phenotype already described in *C. auris* species, forming large aggregates of cells that are very difficult to disintegrate. First, the *in vitro* activity of these drugs was evaluated both in monotherapy and in combination, and results showed synergistic interactions between amphotericin B and echinocandins in most cases, although higher minimum inhibitory concentration (MIC) values were observed against the aggregative isolate compared to non-aggregative counterparts. The most promising drug combinations were then selected for *in vivo* activity evaluation using a *Caenorhabditis elegans* model of candidiasis and by determination of survival rates. The combination of amphotericin B and caspofungin showed the strongest protective effect during *C. elegans* infection with *C. auris* blood isolates, achieving up to 99% *C*. *elegans* survival. Although the MIC values for micafungin were low *in vitro*, these drug concentrations had no effect *in vivo*, and the combination of amphotericin B and micafungin was the least effective. The results of this study showed that the combination amphotericin B and echinocandins might be a promising therapeutic approach for the treatment of invasive candidiasis caused by *C. auris*. Moreover, the nematode *C. elegans* is a suitable alternative model for screening of new therapeutic agents capable of overcoming multidrug-resistant *C. auris* infections.

**IMPORTANCE:**

Multidrug resistance is a rising problem among non-*Candida albicans* species, such as *Candida auris*. This therapeutic problem has been very important during the COVID-19 pandemic. The World Health Organization has included *C. auris* in its global priority list of health-threatening fungi, to study this emerging multidrug-resistant species and to develop effective alternative therapies. In the present study, the synergistic effect of the combination of amphotericin B and echinocandins has been demonstrated against blood isolates of *C. auris*. Different susceptibility responses were also observed between aggregative and non-aggregative phenotypes. The antifungal activity of these drug combinations against *C. auris* was also demonstrated in the *Caenorhabditis elegans* host model of candidiasis, confirming the suitability and usefulness of this model in the search for solutions to antimicrobial resistance.

## INTRODUCTION

*Candida auris* is an emerging multidrug-resistant pathogen first described in 2009 (1, 2) that predominately colonizes the skin, and the invasive infections cause high mortality (30%–72%) (2 – 4). *C. auris* shows a high capacity to develop resistance to commonly used antifungal drugs; to form biofilms; to produce hydrolytic enzymes such as phospholipases, acidic proteases, or hemolysis; and to evade neutrophil attack. These virulence factors contribute to its persistence in the hospital environment and to cause invasive candidiasis (5, 6).

The ability to grow in large cell aggregates is an interesting phenotypic characteristic of some *C. auris* isolates (1). Aggregative isolates are associated with lower pathogenicity but higher antifungal resistance than non-aggregative counterparts (1, 7 – 9). There is a wide genomic diversity of *C. auris*, comprising at least five different clades with different geographical locations, as well as high clonality of *C. auris* isolates within each clade, supporting the hypothesis of a simultaneous and independent emergence of this species in different parts of the world (2, 10, 11).

Noteworthy, many of the clinical isolates of *C. auris* infections have pronounced and sometimes untreatable clinical drug resistance to all available antifungal drugs (12). In this multidrug- and/or pan-resistant pathogen, resistance to fluconazole and amphotericin B (AmB) is common, and resistance to echinocandins is emerging mainly in those countries where they are available; as drug resistance in *C. auris* can be both intrinsic and acquired, prior antifungal use is a risk factor (13). Resistance of *C. auris* to fluconazole can reach up to 100% in outbreaks, although resistance levels vary markedly between clades (12 – 14). Each clade has independently developed azole resistance; while susceptible isolates have been reported especially within the East Asian and South American clades, high resistance rates have been associated with the South Asia clade (13). Resistance to AmB is not as common as resistance to fluconazole, but 30% (10, 13) and up to 40% (15, 16) of isolates have been shown to be resistant, making *C. auris* one of the few fungal species with such high levels of AmB resistance (12). On the other hand, echinocandin-resistant isolates of *C. auris* are not so common, and due to the low incidence of resistance to echinocandins, some authors have proposed these drugs as the most appropriate treatment against *C. auris* infections (2, 17). However, therapeutic failures have been described in patients with candidemia treated with AmB and echinocandins in monotherapy (18, 19). The use of echinocandins as the first-line therapy for invasive *Candida* infections, including *C. auris*, increases the probability of generating resistance, so it is likely to be an acquired trait (12, 13). Resistance to echinocandins has also been reported as clade or isolate dependent (12, 20). In recent years, a notable increase in resistant isolates has been reported in the USA, which evidences a high transmissibility that endangers healthcare environments (21). The effective use of antifungal drugs in combination, mainly echinocandins with AmB or isavuconazole, for *C. auris* candidemia has also been reported in several clinical cases (17, 22). However, these combinations have not been always successful (3), providing contradictory results that need to be clarified to be able to recommend therapy with such drug combinations.

Most antifungal susceptibility studies of *C. auris* have been performed *in vitro*, and only few, with *in vivo* models of candidiasis, the murine model being the most commonly used (5, 7, 11, 15, 23, 24). However, to avoid the ethical restrictions related to the use of mammalian models, the use of invertebrate animal models to study candidiasis has been promoted (1, 7, 8, 25). The nematode *Caenorhabditis elegans* is a widely used alternative host model of candidiasis because of its simplicity, the possibility of using a very large number of individuals in each assay, and the rapid achievement of results. As such, it is a suitable non-mammalian host model for studying the virulence of *Candida* spp., including *C. auris* (8, 26 – 31), as well as the efficacy of antifungal drugs and new antifungal compounds as treatments for candidiasis (25).

In this study, we aimed to analyze the *in vitro* and *in vivo* activities of the combination of AmB and echinocandins against five *C. auris* blood isolates, one of them with an aggregative phenotype. *In vitro* antifungal susceptibility testing was performed using AmB and echinocandins in monotherapy and combining AmB with each echinocandin. The most effective treatments obtained *in vitro* were tested *in vivo* using the *C. elegans* model, and the efficacy of monotherapy and combination treatments against *C. auris* infection was compared.

## RESULTS

### *In vitro* susceptibility of *C. auris* blood isolates to AmB and echinocandins

Antifungal susceptibility of the five *C. auris* blood isolates, with aggregative and non-aggregative phenotypes, to AmB and the echinocandins anidulafungin (AND), caspofungin (CAS), and micafungin (MCF) is shown in Table 1. All isolates were susceptible to AmB, with minimum inhibitory concentration (MIC) values between 0.5 and 1 µg/mL. The four isolates with a non-aggregative phenotype were susceptible to echinocandins; the MIC for CAS was 0.25 µg/mL, and for AND and MCF, the MIC values ranged from 0.12 to 0.25 µg/mL. Strikingly, the *C. auris* isolate with the aggregative phenotype, JMRC:NRZ 1101, was resistant to echinocandins, with MIC values ≥4 µg/mL.

**TABLE 1:** *In vitro* antifungal activity of AmB and echinocandins (AND, CAS, and MCF) against five *C. auris* blood isolates^*a*^

*C. auris* isolate	Source	Phenotype	MIC^*b*^ (µg/mL)	MIC^*c*^ (µg/mL)		
			AmB	AND	CAS	MCF
CJ94	Hospital La Fe (Spain)	Non-aggregative	0.5	0.25	0.25	0.12
CBS 15605	Westerdijk Fungal Biodiversity Institute (via Hospital La Fe, Spain)	Non-aggregative	0.5	0.12	0.25	0.12
CBS 15606	Westerdijk Fungal Biodiversity Institute (via Hospital La Fe, Spain)	Non-aggregative	0.5	0.12	0.25	0.12
CBS 15607	Westerdijk Fungal Biodiversity Institute (via Hospital La Fe, Spain)	Non-aggregative	0.5	0.12	0.25	0.12
JMRC:NRZ 1101	Institut für Hygiene und Mikrobiologie (Germany) Jena Microbial Resource Collection	Aggregative	1	4	>8	4

^*a*^ The *C. auris* JMRC:NRZ 1101 isolate displays an aggregative phenotype, as described previously (7).

^*b*^ MIC, minimal inhibitory concentration of 90% inhibition of cell growth.

^*c*^ MIC, minimal inhibitory concentration of 50% inhibition of cell growth.

To analyze the efficacy of antifungal combinations, MIC values that caused ≥90% inhibition of yeast growth were used for AmB and echinocandins. Interestingly, the combination of AmB and echinocandins resulted in values that were lower than those in monotherapy (Table 2). For AmB, these values were lower for the non-aggregative *C. auris* isolates (ranging from 0.03 to 0.06 µg/mL) than the aggregative isolate (0.25 to 0.5 µg/mL). Regarding the echinocandins, the combination with AmB also resulted in a lowering of the MIC values against the five blood isolates. Notably, for the echinocandins, the MIC in monotherapy was ≥8 µg/mL in all cases. However, for all five isolates, the MIC values were not higher than 2 µg/mL when AmB was combined with AND (0.5–2 µg/mL) and MCF (0.252 µg/mL) and when the combination of AmB plus CAS (0.5–2 µg/mL) was tested against the non-aggregative isolates. For the aggregative isolate, the combination of AmB and CAS resulted in an MIC of 4 µg/mL.

**TABLE 2:** *In vitro* antifungal activity of AmB in combination with echinocandins (AND, CAS, and MCF) against five *C. auris* blood isolates^*a*^

*C. auris* isolate	MIC (µg/mL)	FICI	Bliss	MIC (µg/mL)	FICI	Bliss	MIC (µg/mL)	FICI	Bliss
	AmB AND AmB/AND	Median effect	∑SYN_ANT (∑SYN; ∑ANT)	AmB CAS AmB/CAS	Median effect	∑SYN_ANT (∑SYN; ∑ANT)	AmB MCF AmB/MCF	Median effect	∑SYN_ANT (∑SYN; ∑ANT)
CJ94	0.5 > 8 0.03/1	0.122 S	70.16 (73.55; −3.39)	0.5 > 8 0.03/1	0.122 S	102.71 (108.35; −5.64)	0.5 > 8 0.03/0.25	0.076 S	102.75 (103.04; −0.29)
CBS 15605	0.5 > 8 0.03/0.5	0.091 S	67.71 (67.92; −0.21)	0.5 > 8 0.03/0.5	0.091 S	109.28 (109.90; −0.61)	0.5 > 8 0.03/0.5	0.091 S	96.55 (97.00; −0.45)
CBS 15606	0.5 > 8 0.06/2	0.245 S	40.41 (46.83; −6.42)	0.5 > 8 0.06/1	0.128 S	8.35 (22.34; −30.69)	0.5 > 8 0.06/2	0.245 S	64.22 (65.45; −1.23)
CBS 15607	0.5 > 8 0.03/1	0.122 S	46.47 (48.15; −1.68)	0.5 > 8 0.03/1	0.122 S	74.53 (75.23; −0.71)	0.5 > 8 0.06/0.5	0.151 S	41.02 (44.11; −3.09)
JMRC:NRZ 1101	1 > 8 0.25/2	0.375 S	74.18 (75.08; −0.90)	1 > 8 0.25/4	0.5 S	25.08 (45.25; −20.16)	1 > 8 0.5/0.5	0.562 Ad	49.14 (56.08; −6.94)
GM	0.574 > 8 0.053/1.148	–	–	0.574 > 8 0.054/1.148	–	–	0.574 > 8 0.071/0.574	–	–
Range	0.5–1 > 8 0.03–0.25/0.5–2	–	–	0.5–1 > 8 0.03–0.25/0.5–4	–	–	0.5–1 > 8 0.03–0.5/0.25–2	–	–

^*a*^ Given are the MIC of 90% inhibition of cell growth, fractional inhibitory concentration index, and interaction parameters determined by the Bliss model. FICI, fractional inhibitory concentration index; effect of the interaction: S = synergistic interaction; Ad = additive interaction. Bliss model **∑**SYN_ANT: total sum of synergic and antagonistic interactions; **∑**SYN: sum of synergic interactions; **∑**ANT: sum of antagonistic interactions. GM, geometric mean; MIC, minimal inhibitory concentration of 90% inhibition of cell growth.

^*b*^ –, no value for that parameter.

The combinations of AmB with each of the echinocandins exhibited synergistic interactions against *C. auris* isolates with fractional inhibitory concentration index (FICI) values ≤0.5, between 0.076 and 0.5 (Table 2), except for the combination of AmB and MCF, which showed an additive interaction against the aggregative *C. auris* isolate (JMRC:NRZ 1101) because its FICI value was >0.5 and ≤1 (FICI = 0.562).

Although the FICI method is widely accepted and a good method for its preliminary assessment of synergistic activities of antifungal drug combinations, it is often beneficial to use multiple methods in parallel to obtain a more rounded view of potential drug interactions. In this line, conducting FICI method and Bliss independence model in tandem offered a more comprehensive analysis of drug interactions. In addition, results have been cross-verified with both approaches. The Bliss independence model can offer better predictive power, especially in the case of drugs with independent mechanisms of action, as is the case with AmB and echinocandins. The interaction parameter ∑SYN_ANT, that is the total sum of synergic (**∑**SYN) and antagonistic interactions (**∑**ANT), obtained using the Bliss independence-based model, showed weak synergistic interactions (values less than 100%) for all combinations and isolates, except for AmB plus CAS against isolates CJ94 and CBS 15605 and AmB plus MCF against isolate CJ94, which showed moderate synergistic interactions (values between 100% and 200%).

**Fig 1:**
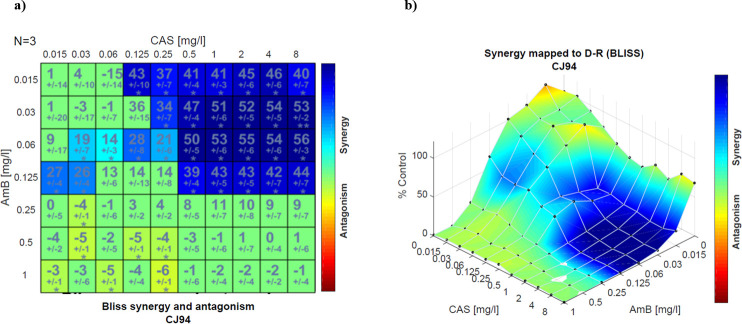
Synergy distribution determined by the Bliss interaction model for the combination of AmB and CAS against *C. auris* CJ94. (a) Matrix synergy plot with synergy scores for each combination. (b) Synergy distribution mapped to dose-response surface.

According to these results, the most active drug combination against *C. auris* was the combination of AmB and MCF, as the MIC values for this combination were equal or lower than those with the other two echinocandins. The geometric mean (GM) of the MICs obtained for AmB plus MCF was 0.574 µg/mL, while for AmB in combination with AND and CAS, it was 1.148 µg/mL. Moreover, Bliss analysis showed that with the combination containing lower AmB (≤0.125 mg/L) and higher echinocandins (≥0.06 mg/L) concentrations, both synergy and a low absorbance value effect were achieved. Figure 1 shows the surface response according to the Bliss method of a representative non-aggregative isolate and drug combinations in a checkerboard design with the blue color representing the synergistic distribution and the color intensity the degree of synergism.

### Antifungal therapy efficacy during *Caenorhabditis elegans* infection with *Candida auris*

**Fig 2:**
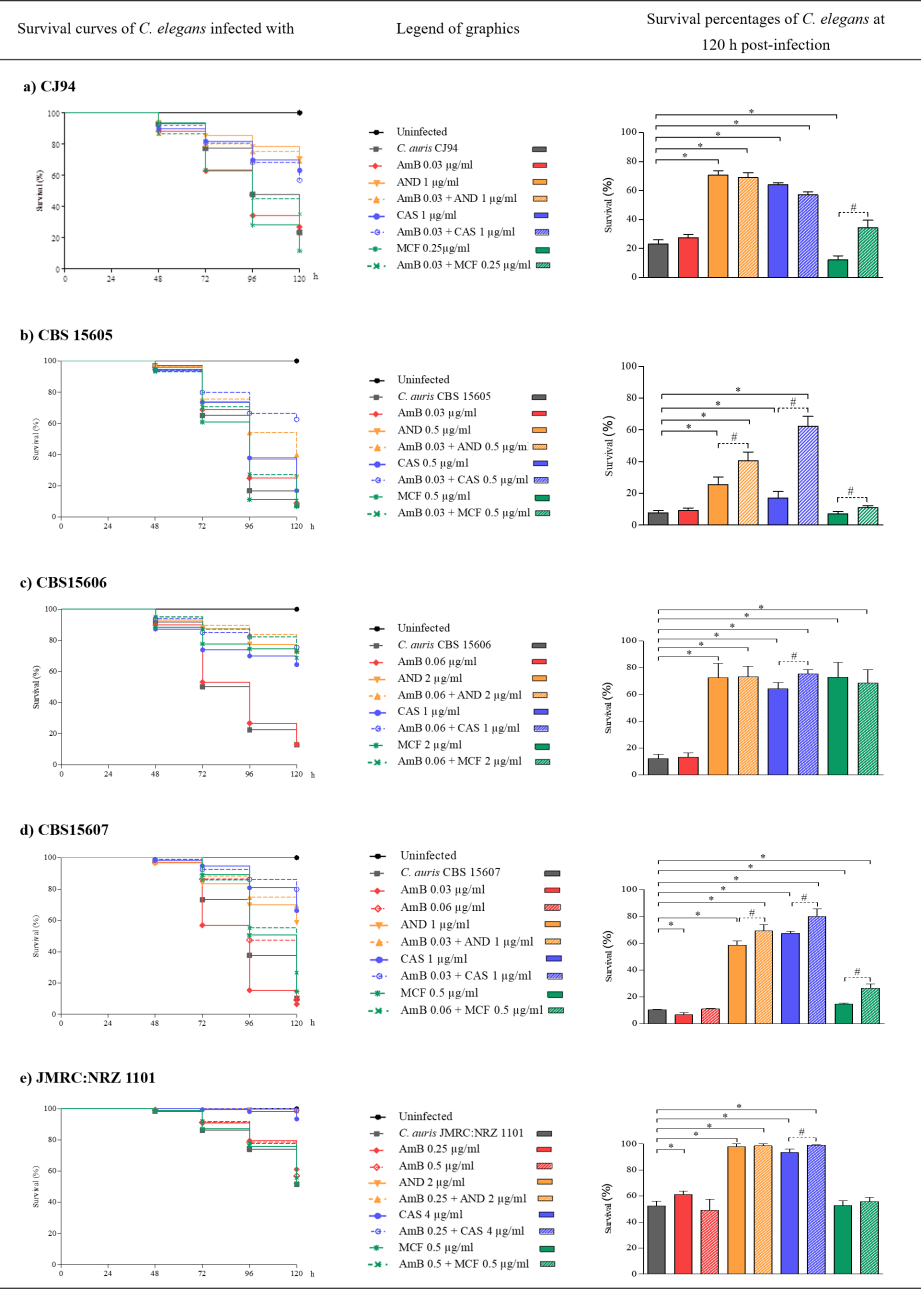
Survival curves and survival percentages at 120 h post-infection of *C. elegans* infected with *C. auris* blood isolates CJ94 (a), CBS 15605 (b), CBS 15606 (c), CBS 15607 (d), and JMRC:NRZ 1101 (e) and treated with the combinations AmB plus AND, AmB plus CAS, and AmB plus MCF. The drug concentrations used for each strain were the MIC values obtained *in vitro* in combination. Statistically significant differences between survival of infected *C. elegans* nematodes in the presence of drug combinations and infected but untreated (*) or infected and treated with the corresponding echinocandins in monotherapy (#) are indicated. No significant differences were detected between nematodes in the presence of drugs used to analyze their toxicity and nematodes in the control groups, as the survival in all cases was 100% (*P* > 0.05).

The antifungal activity of the combination of AmB and echinocandins was assessed in the *C. elegans in vivo* model of candidiasis. The drug concentrations tested *in vivo* for each isolate, both in monotherapy and in combination, were the MIC values obtained *in vitro* in the combination assays. The drugs studied were not toxic to the nematodes neither in monotherapy nor in combination, since the survival rate after 120 h was 100%, with no significant differences with the control groups. The five *C. auris* blood isolates were able to kill the nematode (7.6%–52.3% survival), while uninfected nematodes used as controls remained viable (100% survival) during the 120 h post-infection. The efficacy of the antifungal drugs *in vivo*, determined as prolonged survival of *C. auris*-infected nematodes, is detailed in Fig. 2 and Table S1.

Treatment with AmB, as expected due to the low concentration tested compared to the MIC of this drug *in vitro* monotherapy assays, did not significantly prolong the survival of infected nematodes compared to untreated ones, except for nematodes infected with isolate JMRC:NRZ 1101 treated with 0.25 µg/mL AmB. However, treatment of the nematodes with echinocandins (AND and CAS) alone and in combination with AmB resulted in significantly improved efficacy against *C. auris* infection in an isolate-dependent manner. That is, the treatments tested at the isolate-specific drug concentrations were able to reduce nematode mortality in some, but not all, isolate infections. For example, for isolates CBS 15605 and CBS 15607, treatment with the combination of AmB and echinocandins resulted in significantly higher survival of infected nematodes than for the other isolates.

Treatment with a combination of AmB and CAS, at the concentrations tested, was the most effective, reaching survival rates in *C. elegans* ranging from 56.8% to 99.0% and a mean mortality reduction of 53.5% compared to untreated nematodes. CAS used in monotherapy also had a protective effect during *C. elegans* infection, reducing nematode mortality between 9.4% and 56.9%. The combination of AmB and CAS significantly reduced the *C. elegans* mortality compared to monotherapy (*P* ≤ 0.05), except for the infection with isolate CJ94 that showed no significant improvement in survival for CAS in combination with AmB compared to CAS monotherapy (survival of 66.6% versus 60.3%, respectively) (Fig. 2a).

AND was also effective against *C. auris* infection compared to untreated nematodes. Infected with isolates CBS 15605 and CBS 15607, the combination of AmB and AND provided better protection to *C. elegans* than AND alone (*P* ≤ 0.05) (Table S1). However, for the remaining *C. auris* isolates studied, the combination treatment, using the concentrations according to the *in vitro* results, did not significantly improve survival over AND alone. The combination of AmB and AND achieved *C. elegans* survival ranging from 39.9% to 98.8%, and the mean reduction in nematode mortality was 48.8% compared to untreated nematodes. For strain CJ94, this combination was the most active (45.6% mortality reduction), although there was no significant difference with AND alone (47.1% mortality reduction). In the aggregative isolate JMRC:NRZ 1101, the efficiency of the combination of AmB and AND was comparable to the combination of AmB and CAS. However, when infected with isolates CBS 15605 and CBS 15607, the combination of AmB and CAS resulted, based on the log-rank analysis, in higher survival rates of *C. elegans* than with the combination of AmB and AND (*P* ≤ 0.022). Furthermore, an equal or lower concentration of CAS combined with AmB was more or equally effective than AND combined with AmB against *C. elegans* infection with non-aggregative *C. auris* isolates (Fig. 2).

MCF, at the concentrations tested, was the least effective echinocandin against *C. auris* infection. MCF alone and combined with AmB only showed significant activity during *C. elegans* infection with *C. auris* CBS 15606 (*P* ≤ 0.0001) and CBS 15607 (*P* ≤ 0.0001) isolates compared to untreated *C. elegans* (Table S1). The survival rate of *C. elegans* infected with CBS 15606 increased up to 68.7% with this drug combination. However, the mean reduction in nematode mortality infected with the five *C. auris* blood isolates was only 18.1% due to the reduced activity against the aggregative isolate (JMRC:NRZ 1101) and three non-aggregative isolates (CJ94, CBS 15605, and CBS 15607). Overall, although fungal growth inhibition was achieved at the same concentrations in the *in vitro* test, the combination of AmB plus MCF was significantly less effective *in vivo* than the other two combinations of echinocandins plus AmB (*P* ≤ 0.0001) (Fig. 2).

## DISCUSSION

*C. auris* has become a dangerous emerging pathogen worldwide due to its ability to form biofilms on medical devices, efficient skin colonization, persistence in the hospital environment for weeks or even months, evasion of neutrophil attack, and development of multiple resistance to commonly used antifungal drugs (2, 4 – 6). This situation has aggravated with the COVID-19 pandemic. Chowdhary et al. reported bloodstream infections caused by multidrug-resistant *C. auris* in COVID-19 intensive care unit patients in India, with *C. auris* being the predominant agent in those cases suffering invasive candidiasis (16). Recently, Vaseghi et al (32) conducted a review and meta-analysis of the global prevalence of coronavirus-associated *C. auris* infection, suggesting that further studies are needed to determine the exact cause-effect relationships between *C. auris* and COVID-19. The authors concluded that the prevalence of *C. auris* infections in the COVID-19 population is lower than in other critically ill patients at the pre-COVID era. However, mortality rates in patients with *C. auris* infection increased in the COVID-19 era, and fluconazole resistance continues to be the highest antifungal resistance after COVID-19 pandemic (32). The limited antifungal therapeutic options against *C. auris* infection are an unresolved and highly concerning challenge that requires attention (4, 12, 33).

*C. auris* is highly resistant to fluconazole (2, 15, 34) and shows a reduced susceptibility to AmB. Chowdhary et al. (15) observed AmB resistance in 8% of *C. auris* isolates, while Tsay et al. (35) reported 43% of resistant isolates. However, most authors described AmB resistance levels between these two values (2, 15, 16, 36). Echinocandins, the first-line treatment currently recommended for most invasive candidiasis, are active in most studies, with between 2% and 3% of resistant *C. auris* isolates being reported (15, 35) or even as low as 1% of resistant *C. auris* isolates in a New York outbreak from 2016 to 2018 (34). In the present study, *in vitro* results showed the efficacy of AmB and echinocandins, except against the *C. auris* isolate with an aggregative phenotype. This aggregative isolate was resistant to all echinocandins (MIC ≥4 µg/mL), and the MIC value of AmB was the highest of all (1 µg/mL versus 0.5 µg/mL in the other isolates). Previous studies have reported higher MIC values for AmB against *C. auris* (GM 0.94 µg/mL) (37) than those obtained in the present study, although many studies are in concordance with the results of our study (36, 38). Conversely and similar to our findings, MIC values of ≤1 µg/mL for AmB against isolates from the four main clades of *C. auris* were recently reported (39). Interestingly, these authors found weak lethal activity of AmB against all *C. auris* clades in time-kill studies, even at low MIC values (1 µg/mL), suggesting that the efficacy of this drug against *C. auris* may be unpredictable (39). This background supports the interest in performing *in vivo* studies, such as the one developed in the present work, as well as the need for combination therapy studies in both *in vitro* and *in vivo* models.

Regarding echinocandins, we found lower MIC values than those observed for AmB, in line with previously reported *in vitro* results (36, 37). High MIC values for echinocandins against *C. auris* isolates have been reported in an outbreak in the UK (40), in different clinical studies from the USA (41, 42), and in a previous study from India, where high MIC values for CAS were reported for 37% of *C. auris* isolates, 24% of which were resistant (≥2 µg/mL) (43). Interestingly, reversible induction of the aggregative phenotype was reported in isolates of the South Asian lineage exposed to triazoles or echinocandins, which may be an additional problem in the treatment of *C. auris* infection (44). Kovacs et al. also reported that echinocandins induced large aggregates, which may explain the weak inhibition without fungicidal activity against *C. auris* that is not reflected in the obtained MIC values (45).

Combination therapies are a promising strategy against candidiasis caused by multidrug-resistant *Candida* species. However, scarce evidence exists about antifungal combinations against *C. auris* infection. Few clinical trials have reported outcomes in patients with *C. auris* candidemia treated with echinocandins in combination with AmB (3, 17, 46) or isavuconazole (22). In addition, relatively few *in vitro* studies have analyzed the efficacy of antifungal compound combinations against *C. auris* (47 – 56). In these studies, synergy was achieved against *C. auris* with the combination of MCF plus voriconazole, but indifference was observed with the combination of CAS plus voriconazole (47). A synergistic effect was also obtained with the combination of AND plus voriconazole and AND plus isavuconazole against 14% and 31%, respectively, of the *C. auris* isolates analyzed (37). In other studies, the combination of isavuconazole with echinocandins, analyzed with different *in vitro* approaches, also achieved synergism and fungistatic activity against *C. auris* (24, 52, 55). In addition, the interaction of echinocandins (AND and CAS) with flucytosine or with the non-antifungal drug colistin presented synergistic effects, while in combination with MCF, these two compounds showed indifferent effects against *C. auris* (48 – 50). The combination of AmB and echinocandins (AND and CAS) against *C. auris* has been previously evaluated by *in vitro* time-kill studies and pharmacokinetic/pharmacodynamic (PK/PD) modeling and simulation approaches, with synergistic and fungicidal results (53). Compared to monotherapy, our study showed improved antifungal activity when these drugs were combined, and the achievement of synergy was related to higher concentrations of echinocandin in the combination, consistent with results reported by Caballero et al. (53). Furthermore, we detected different interaction outcomes depending on the aggregative or non-aggregative nature of the *C. auris* isolates. With regard to the aggregative isolate JMRC:NRZ 1101, the *in vitro* interaction of AmB plus MCF was additive, whereas the combination with the other two echinocandins was classified as synergistic. On the other hand, the combination of AmB and echinocandins resulted in a synergistic interaction against all non-aggregative isolates *in vitro*, with the combination of AmB and MCF being the most active. In contrast, O’Brien et al. (50) did not find synergism for the combinations of AmB and echinocandins when testing activity against *C. auris* isolates from a New York outbreak. Those strains were related to the South Asian clade (50), whereas those in the present study are phylogenetically close to clade III isolates (South African clade) (10, 19). These findings highlight differences, probably related to the phenotype as well as genotype, within the species *C. auris*. This fact has already been noted both in antifungal drug interactions (52) as well as in virulence (8) and in the genomic diversity of this species (10). In this line, one of the limitations of the present study was that genetically diverse isolates were not included. In future studies, the inclusion of additional isolates, especially from different clades of *C. auris*, would expand the knowledge and application of the results of this work.

The synergistic effect for AmB and echinocandins observed *in vitro* was further assessed *in vivo*, using the *C. elegans* host model, to establish a possible correlation. The nematode *C. elegans* has been shown to be useful as host model of *Candida* infection to increase knowledge of the virulence and the efficacy of treatments for candidiasis caused by different *Candida* species (25, 57), including *C. auris* (8, 26 – 31). Concretely, combination therapy studies of *C. elegans* infected with *C. auris* have been conducted to evaluate the combination of azoles with other drugs, such as sulfamethoxazole (26), the HIV protease inhibitor lopinavir (27), the antiemetic agent aprepitant (28), and the stilbene compound ospemifene (29). All these combination treatments were reported to enhance the survival of *C. elegans* compared to those treated with the respective monotherapy.

This study confirmed, for the first time, the efficacy of antifungal combination therapies with AmB and echinocandins both *in vitro* and *in vivo* in a *C. elegans* model of *C. auris* infection. However, the *in vivo* results were not always as effective as those observed *in vitro*, and there were differences between the combinations of AmB with each of the echinocandins, challenging whether the concentration obtained *in vitro* will always be as effective *in vivo*. Our results identified the combination of AmB plus CAS as the most effective when testing the MIC values obtained *in vitro* for each *C. auris* isolate, as higher nematode survival during infection was achieved by four of the five clinical blood isolates. The effect of AmB plus CAS was similar to AmB plus AND against infection with the aggregative isolate JMRC:NRZ 1101, yielding nematode survival of nearly 100% (99.0% and 98.8%, respectively). In monotherapy, these drugs also provided *C. elegans* protection against *C. auris* infection. Strikingly, the combination of AmB and MCF and these drugs in monotherapy were the least effective and, in some cases, ineffective during *C. elegans* infection with *C. auris*. However, in a retrospective cohort study of neonates suffering from *C. auris* sepsis, the treatment with a combination of AmB and MCF was effective, as patient survival increased to 83% (58). Therefore, although MCF was the echinocandin that required the lowest concentrations *in vitro* to inhibit the growth of the isolates, these concentrations were not effective *in vivo*. Perhaps, in future studies, a similar concentration should be used for the three echinocandins, for instance, the highest or intermediate concentration range detected *in vitro*. The differences in antifungal drug therapies found between *in vitro* and *in vivo* assays highlight the importance of confirming the *in vitro* results using *in vivo* systems due to their complexity. The non-mammalian host *C. elegans* has once again proved its usefulness as model of candidiasis to perform initial *in vivo* approaches.

To conclude, this study demonstrates the synergistic effect *in vitro* and *in vivo* of AmB and echinocandin combinations against *C. auris*. Variations in antifungal activity were observed, possibly related to the ability of *C. auris* to form cell aggregates. The isolate with the aggregative phenotype showed higher *in vitro* MIC values compared to the non-aggregative isolates both in monotherapy and in combination assays. Interestingly, it has been suggested that the formation of aggregates may be a survival strategy of *C. auris* (20), which has also been observed *in vivo* (59). Based on our findings, when MIC values of antifungal susceptibility testing are high in monotherapy for a *C. auris* isolate, combination therapy has been shown to be a good alternative to reduce drug concentrations. Overall, the combination of AmB and CAS was the most effective in reducing mortality of *C. elegans* upon *C. auris* infection, while the combination of AmB and MCF was the least effective. The efficacy of the combination of AmB and echinocandins against *C. auris in vitro* and in the *C. elegans* host model supports combination therapy as a promising tool against *C. auris* infection and warrants further studies in this regard.

## MATERIALS AND METHODS

### *Candida auris* isolates and growth conditions

Five clinical *C. auris* blood isolates were used in this study (Table 1). Among them, the four with non-aggregative phenotype were obtained from the Hospital Universitario y Politécnico La Fe of Valencia, Spain, three of them being registered in the CBS-KNAW culture collection of the Westerdijk Fungal Biodiversity Institute. The remaining isolate, *C. auris* JMRC:NRZ 1101 from the Jena Microbial Resource Collection, was provided by the Institut für Hygiene und Mikrobiologie, Würzburg, Germany. This latter *C. auris* isolate exhibits an aggregative phenotype (8).

Isolates stored cryopreserved at −80°C were recovered on Sabouraud dextrose agar (Difco, Becton Dickinson, USA) and incubated at 37°C for 24 h. For the assays with the *C. elegans* model, brain-heart infusion (BHI) (Panreac, Spain) agar plates supplemented with kanamycin (90 µg/mL) were seeded with a cell suspension of 2 McFarland of each *C. auris* isolate and incubated at 37°C for 24 h.

### Determination of *in vitro* antifungal drug activity

The antifungal drugs tested were AmB (Sigma-Aldrich, Inc., USA), AND (Pfizer SA, Spain), CAS (Merk and Com, Inc., USA), and MCF (Astellas Pharma, Inc., Japan). Stock solutions were prepared in dimethyl sulfoxide (DMSO) for *in vitro* and *in vivo* studies.

The antifungal activity of each drug was determined by microdilution antifungal susceptibility testing as described in the European Committee on Antimicrobial Susceptibility Testing document E.DEF 7.3, using 96-well microtiter plates. Drug concentrations tested ranged from 0.03 to 16 µg/mL for AmB and from 0.016 to 8 µg/mL for echinocandins. Briefly, *C. auris* inocula were prepared in distilled water, and 200 µL of a 0.5 McFarland standard yeast suspension was transferred into 1.8 mL of distilled water tubes. Then, each well containing 100 µL of antifungal drug in 2× RPMI 1640 medium was mixed with 100 µL of the inoculum. The microtiter plates were incubated at 37°C for 24 h, and growth was measured at a wavelength of 450 nm using a spectrophotometer (Tecan, Switzerland). As described in the protocol, the MIC was considered as the lowest concentration that caused ≥50% for echinocandins and ≥90% for AmB inhibition of yeast growth compared to the growth without antifungal drug. The susceptibility of isolates was categorized according to the provisional breakpoints proposed by the CDC: AmB (≥2 µg/mL) and echinocandins (≥4 µg/mL for AND and MCF; ≥2 µg/mL for CAS).

The antifungal drug efficacy was also tested *in vitro* for the following drug combinations: AmB plus AND, AmB plus CAS, and AmB plus MCF. Combination assays were assessed by the broth microdilution checkerboard method using 96-well flat-bottom microtiter plates (60). Drug concentrations assayed ranged from 0.015 to 1 µg/mL for AmB and from 0.016 to 8 µg/mL for each echinocandin. Briefly, yeast cell suspensions adjusted to a 0.5 McFarland turbidity standard of each *C. auris* isolate were prepared in distilled water and diluted 10-fold in RPMI 1640 medium. In addition, 50 µL of each dilution of AmB was added vertically to each well of a microtiter plate, and the same amount of each dilution of each echinocandin was added horizontally to obtain the different antifungal combinations. Subsequently, 100 µL of each yeast inoculum was mixed with 100 µL of antifungal drugs in each well. Wells from a vertical column were left for growth control, and others from a horizontal row were used as sterility control. After incubation of the microtiter plates at 37°C for 24 h, yeast growth was measured with a spectrophotometer at a wavelength of 450 nm. Absorbance data were normalized, with the mean absorbance of the growth control set at 100%. Antifungal drug interactions were evaluated using the FICI method and by the Bliss independence as a surface response model. The FICI is based on Loewe’s additivity, and the values obtained for the MIC of 90% inhibition of cell growth were applied to the formula FICI = MIC_A/E_/MIC_A_ + MIC_E/A_/MIC_E_ (MIC_A/E_ = MIC for AmB in combination; MIC_A_ = MIC for AmB in monotherapy; MIC_E/A_= MIC for echinocandins in combination with AmB; MIC_E_ = MIC for echinocandins in monotherapy). Interactions between antifungal drugs were classified as synergistic when FICI ≤0.5, additive when 0.5 ˂ FICI ≤ 1, indifferent when 1 < FICI ≤ 4, and antagonistic when FICI >4 (60). The Bliss independence model, which assumes that the relative effect of a drug at a particular concentration is independent of the other drug, was performed using Combenefit software (61). This model calculates the difference between the predicted percentage of growth (Eind) and the observed percentage of growth (Eobs): ∆E = Eind ­− Eobs. The value of Eind is obtained from the equation Eind = EA × EB, where EA and EB are the observed percentages of growth in the presence of drug A and drug B, respectively. Interactions were classified as synergistic when the ∆E of each specific combination of *x* mg/L of AmB and *y* mg/L of echinocandin was positive and the 95% confidence interval (CI) did not include zero, as antagonistic when the ∆E was negative and its 95% CI did not include zero, and as indifferent for the other cases. The software shows the sum of all statistically significant synergistic and antagonistic interactions (**∑**SYN_ANT) for each checkerboard analysis, and this parameter was used to summarize the whole interaction surface for the three combinations studied. Weak interaction was defined when the **∑**SYN_ANT value was below 100%; moderate, when it was between 100% and 200%; and strong, when the value was higher than 200% (62, 63).

### *In vivo* assays of antifungal combination in *Caenorhabditis elegans*

The *C. elegans* AU37 double-mutant strain (*glp-4*(*bn2*); *sek-1*(*km4*)) obtained from the Caenorhabditis Genetics Center (University of Minnesota, USA) was used to perform the *in vivo* assays. These mutations render the nematodes sterile at 25°C (*glp-4*) and more susceptible to infection (*sek-1*), thus ensuring a constant number of individuals throughout the experiments.

Survival studies were performed using a synchronous population of *C. elegans* at the L4 larval stage as previously described (57). Nematodes were placed at 25°C for 2 h onto BHI agar plates supplemented with kanamycin (90 µg/mL) seeded with lawns of the clinical *C. auris* isolates to ingest them. After that, nematodes were washed with M9 buffer (3 g of KH_2_PO_4_, 6 g of Na_2_HPO_4_, 5 g of NaCl, 1 mL of 1 M MgSO_4_ and H_2_O to 1 L) supplemented with kanamycin (90 µg/mL) and transferred for 15 min to plates with nematode growth medium (3 g of NaCl, 17 g of agar, 2.5 g of peptone, 1 mL of 1 M CaCl_2_, 1 mL of 5 mg/mL cholesterol in ethanol, 1 mL of 1 M MgSO_4_, 25 mL of 1 M KPO_4_, and 975 mL of H_2_O) to remove yeasts adhering to their cuticles. *C. elegans* infected with *C. auris* were then displaced in groups of 20 individuals in 24-well plates, and antifungal compounds (with 0.1% DMSO) were added to the wells both in monotherapy and in combination. The antifungal drug concentrations that gave the best results in the *in vitro* antifungal susceptibility test were tested *in vivo*. Plates were incubated at 25°C for 120 h, and nematode survival was observed every 24 h using a stereomicroscope (Nikon SMZ-745, Japan). Nematodes were considered dead when no movement was detected, and yeast growth was observed inside them. Experiments were performed at least in triplicate in different weeks. In each experiment, seven different treatments were evaluated for each *C. auris* isolate, and 60 nematodes were used in each condition. Moreover, two control groups were used in each experiment: one of uninfected nematodes and another of infected nematodes without antifungal treatment. To assess the toxicity of the compounds, uninfected nematodes were also assayed in the presence of antifungal drugs only, both in monotherapy and in combination. In total, around 15,000 nematodes were assayed in this work.

### Statistics

Kaplan-Meier curves were generated using GraphPad Prism 5 (GraphPad Software, La Jolla, CA, USA) to assess the survival of *C. elegans* during *C. auris* infection and exposure to the different antifungal drug treatments. Survival curves of uninfected nematodes and control ones that were not exposed to drugs were also plotted. Differences in survival of *C. elegans* were analyzed and compared by log-rank test using SPSS v26.0 (IBM, Chicago, IL, USA). Values of *P* < 0.05 were considered as statistically significant.

## SUPPLEMENTAL MATERIAL

Table S1 spectrum.02086-23-s0001.docx Statistical analysis of differences in treatment efficacy of the combination of amphotericin B and echinocandins against *C. auris* blood isolates in *C. elegans.*

ASM does not own the copyrights to Supplemental Material that may be linked to, or accessed through, an article. The authors have granted ASM a non-exclusive, world-wide license to publish the Supplemental Material files. Please contact the corresponding author directly for reuse.
