# Supplementary material for: Efficacy of the combination of amphotericin B and echinocandins against Candida auris in vitro and in the Caenorhabditis elegans host model
Source: Microbiol Spectr. 2023 Nov 29;12(1):e02086-23. doi: 10.1128/spectrum.02086-23 (PMC10783041; doi:10.1128/spectrum.02086-23)
Supplement: Table S1 — Statistical analysis of differences in treatment efficacy of the combination of amphotericin B and echinocandins against C. auris blood isolates in C. elegans. [file spectrum.02086-23-s0001.docx]

**Table S1.** Statistical analysis of differences in treatment efficacy of the combination of amphotericin B (AmB) and echinocandins (anidulafungin, AND; caspofungin, CAS; and micafungin, MCF) against *C. auris* blood isolates in *C. elegans* infected by *C. auris* ingestion for 2 h. The analysis was performed using the log-rank test and values of p ≤ 0.05 were considered statistically significant. The letters ns indicate non-significant differences (p > 0.05).

| ***C. auris* CJ94** | p values | | | | | | | | |
| --- | --- | --- | --- | --- | --- | --- | --- | --- | --- |
|  | Uninfected  larvae | *C. auris* CJ94  infected and untreated | AmB  0.03 µg/ml | AND  1 µg/ml | AmB/AND  0.03/1 µg/ml | CAS  1 µg/ml | AmB/CAS  0.03/1 µg/ml | MCF  0.25 µg/ml | AmB/MCF  0.03/0.25 µg/ml |
| Uninfected larvae |  |  |  |  |  |  |  |  |  |
| *C. auris* CJ94 infected and untreated | 0.0001 |  |  |  |  |  |  |  |  |
| AmB 0.03 µg/ml | 0.0001 | ns |  |  |  |  |  |  |  |
| AND 1 µg/ml | 0.0001 | 0.0001 | 0.0001 |  |  |  |  |  |  |
| AmB/AND 0.03/1 µg/ml | 0.0001 | 0.0001 | 0.0001 | ns |  |  |  |  |  |
| CAS 1 µg/ml | 0.0001 | 0.0001 | 0.0001 | ns | ns |  |  |  |  |
| AmB/CAS 0.03/1 µg/ml | 0.0001 | 0.0001 | 0.0001 | 0.004 | 0.042 | ns |  |  |  |
| MCF 0.25 µg/ml | 0.0001 | 0.0001 | 0.013 | 0.0001 | 0.0001 | 0.0001 | 0.0001 |  |  |
| AmB/MCF 0.03/0.25 µg/ml | 0.0001 | ns | ns | 0.0001 | 0.0001 | 0.0001 | 0.0001 | 0.0001 |  |

| ***C. auris* CBS 15605** | p values | | | | | | | | |
| --- | --- | --- | --- | --- | --- | --- | --- | --- | --- |
|  | Uninfected  larvae | *C. auris* CBS 15605  infected and untreated | AmB  0.03 µg/ml | AND  0.5 µg/ml | AmB/AND  0.03/0.5 µg/ml | CAS  0.5 µg/ml | AmB/CAS  0.03/0.5 µg/ml | MCF  0.5 µg/ml | AmB/MCF  0.03/0.5 µg/ml |
| Uninfected larvae |  |  |  |  |  |  |  |  |  |
| *C. auris* CBS 15605 infected and untreated | 0.0001 |  |  |  |  |  |  |  |  |
| AmB 0.03 µg/ml | 0.0001 | ns |  |  |  |  |  |  |  |
| AND 0.5 µg/ml | 0.0001 | 0.0001 | 0.0001 |  |  |  |  |  |  |
| AmB/AND 0.03/0.5 µg/ml | 0.0001 | 0.0001 | 0.0001 | 0.002 |  |  |  |  |  |
| CAS 0.5 µg/ml | 0.0001 | 0.0001 | 0.009 | ns | 0.0001 |  |  |  |  |
| AmB/CAS 0.03/0.5 µg/ml | 0.0001 | 0.0001 | 0.0001 | 0.0001 | 0.0001 | 0.0001 |  |  |  |
| MCF 0.5 µg/ml | 0.0001 | ns | 0.011 | 0.0001 | 0.0001 | 0.0001 | 0.0001 |  |  |
| AmB/MCF 0.03/0.5 µg/ml | 0.0001 | ns | ns | 0.001 | 0.0001 | 0.039 | 0.0001 | 0.003 |  |

| ***C. auris* CBS 15606** | p values | | | | | | | | |
| --- | --- | --- | --- | --- | --- | --- | --- | --- | --- |
|  | Uninfected  larvae | *C. auris* CBS 15606  infected and untreated | AmB  0.06 µg/ml | AND  2 µg/ml | AmB/AND  0.06/2 µg/ml | CAS  1 µg/ml | AmB/CAS  0.06/1 µg/ml | MCF  2 µg/ml | AmB/MCF  0.06/2 µg/ml |
| Uninfected larvae |  |  |  |  |  |  |  |  |  |
| *C. auris* CBS 15606 infected and untreated | 0.0001 |  |  |  |  |  |  |  |  |
| AmB 0.06 µg/ml | 0.0001 | ns |  |  |  |  |  |  |  |
| AND 2 µg/ml | 0.0001 | 0.0001 | 0.0001 |  |  |  |  |  |  |
| AmB/AND 0.06/2 µg/ml | 0.0001 | 0.0001 | 0.0001 | ns |  |  |  |  |  |
| CAS 1 µg/ml | 0.0001 | 0.0001 | 0.0001 | 0.043 | 0.047 |  |  |  |  |
| AmB/CAS 0.06/1 µg/ml | 0.0001 | 0.0001 | 0.0001 | ns | 0.0001 | 0.015 |  |  |  |
| MCF 2 µg/ml | 0.0001 | 0.0001 | 0.0001 | ns | 0.0001 | ns | ns |  |  |
| AmB/MCF 0.06/2 µg/ml | 0.0001 | 0.0001 | 0.0001 | ns | 0.0001 | ns | ns | ns |  |

| ***C. auris* CBS 15607** | p values | | | | | | | | | |
| --- | --- | --- | --- | --- | --- | --- | --- | --- | --- | --- |
|  | Uninfected  larvae | *C. auris* CBS 15607  infected and untreated | AmB  0.03 µg/ml | AmB  0.06 µg/ml | AND  1 µg/ml | AmB/AND  0.03/1 µg/ml | CAS  1 µg/ml | AmB/CAS  0.03/1 µg/ml | MCF  0.5 µg/ml | AmB/MCF 0.06/0.5 µg/ml |
| Uninfected larvae |  |  |  |  |  |  |  |  |  |  |
| *C. auris* CBS 15607 infected and untreated | 0.0001 |  |  |  |  |  |  |  |  |  |
| AmB 0.03 µg/ml | 0.0001 | 0.0001 |  |  |  |  |  |  |  |  |
| AmB 0.06 µg/ml | 0.0001 | 0.015 | 0.0001 |  |  |  |  |  |  |  |
| AND 1 µg/ml | 0.0001 | 0.0001 | 0.0001 | 0.0001 |  |  |  |  |  |  |
| AmB/AND 0.03/1 µg/ml | 0.0001 | 0.0001 | 0.0001 | 0.0001 | 0.032 |  |  |  |  |  |
| CAS 1 µg/ml | 0.0001 | 0.0001 | 0.0001 | 0.0001 | ns | ns |  |  |  |  |
| AmB/CAS 0.03/1 µg/ml | 0.0001 | 0.0001 | 0.0001 | 0.0001 | 0.0001 | 0.022 | ns |  |  |  |
| MCF 0.5 µg/ml | 0.0001 | 0.0001 | 0.0001 | ns | 0.0001 | 0.0001 | 0.0001 | 0.0001 |  |  |
| AmB/MCF 0.06/0.5 µg/ml | 0.0001 | 0.0001 | 0.0001 | 0.0001 | 0.0001 | 0.0001 | 0.0001 | 0.0001 | 0.024 |  |

| ***C. auris* JMRC:NRZ 1101** | p values | | | | | | | | | |
| --- | --- | --- | --- | --- | --- | --- | --- | --- | --- | --- |
|  | Uninfected  larvae | *C. auris*  JMRC:NRZ 1101  infected and untreated | AmB  0.25 µg/ml | AmB  0.5 µg/ml | AND  2 µg/ml | AmB/AND  0.25/2 µg/ml | CAS  4 µg/ml | AmB/CAS  0.25/ 4 µg/ml | MCF  0.5 µg/ml | AmB/MCF  0.5/0.5 µg/ml |
| Uninfected larvae |  |  |  |  |  |  |  |  |  |  |
| *C. auris* JMRC:NRZ 1101  infected and untreated | 0.0001 |  |  |  |  |  |  |  |  |  |
| AmB 0.25 µg/ml | 0.0001 | 0.016 |  |  |  |  |  |  |  |  |
| AmB 0.5 µg/ml | 0.0001 | ns | ns |  |  |  |  |  |  |  |
| AND 2 µg/ml | 0.0001 | 0.0001 | 0.0001 | 0.0001 |  |  |  |  |  |  |
| AmB 0.25/AND 2 µg/ml | 0.0001 | 0.0001 | 0.0001 | 0.0001 | ns |  |  |  |  |  |
| CAS 4 µg/ml | 0.0001 | 0.0001 | 0.0001 | 0.0001 | 0.032 | 0.01 |  |  |  |  |
| AmB/CAS 0.25/4 µg/ml | 0.0001 | 0.0001 | 0.0001 | 0.0001 | ns | ns | 0.005 |  |  |  |
| MCF 0.5 µg/ml | 0.0001 | ns | 0.013 | ns | 0.0001 | 0.0001 | 0.0001 | 0.0001 |  |  |
| AmB/MCF 0.5/0.5 µg/ml | 0.0001 | ns | ns | ns | 0.0001 | 0.0001 | 0.0001 | 0.0001 | ns |  |
